# Supplementary material for: Complex genetic effects linked to plasma protein abundance in the UK Biobank
Source: Nat Commun. 2025 Dec 14;17:533. doi: 10.1038/s41467-025-67235-0 (PMC12804929; doi:10.1038/s41467-025-67235-0)
Supplement: Supplementary file 1 — Supplementary Information [file 41467_2025_67235_MOESM1_ESM.pdf]

Supplementary Information

Complex genetic effects linked to plasma protein abundance in the UK Biobank

Authors

Arnor I. Sigurdsson, Justus F. Gräf, Zhiyu Yang, Kirstine Ravn, Jonas Meisner, Roman Thielemann, Henry Webel, Roelof A. J. Smit, Lili Niu, Matthias Mann, FinnGen, Bjarni Vilhjalmsson, Benjamin M. Neale, Jens-Christian Holm, Andrea Ganna, Torben Hansen, Ruth J. F. Loos, Simon Rasmussen

Supplementary Note 1: Variant pre-filtering using GWAS

We implemented variant pre-filtering by GWAS into the EIR-auto-GP pipeline to reduce the number of input variants in the DL model to prevent overfitting and reduce computational costs (**Methods**). For each protein, a GWAS using PLINK2[1] was performed to identify pQTL associations. The results of the GWAS were overall concordant with results from Sun et al.[2] despite different input variants (**Figure 1b**). Specifically, while Sun et al., identified 14,287 significant (P<1.7e-11) associations when using 16.1 million variants from imputed data, we identified 172,854 significant (P<1.7e-11) associations among 49,276 unique variants when using 424,097 autosomal variants from called genotypes (**Supplementary Data 1**). We found their distribution across the genome closely matched that of the previous study. For example, pQTLs on chromosomes 6, 9, 10, and 19 were highly associated with protein levels across the whole proteome and overlapping SNVs highly correlated with cor=0.96, pval=2e-308 (**Supplementary Figure 1d, Figure 1b**). The discrepancy in the number of significant hits was likely due to analyzing called genotypes, not applying LD pruning, and that we used a different tool for the GWAS, i.e., PLINK2[1] as opposed to REGENIE[3]. The choice of PLINK2, which employs a simple linear regression approach, could contribute to a higher proportion of false positives compared to REGENIE. REGENIE approximates a mixed model, which is generally more effective at controlling for false positives. Additionally, while Sun et al. used Inverse-rank normal transformed (INT) protein values, we used untransformed NPX values, which might lead to inflation of p-values and an increased number of false-positive associations. Most (67.2%) variants were only associated with a single protein, with some, such as the ABO intron variant rs507666, associating with 249 different protein levels (**Supplementary Figure 1e**). We found most proteins to be associated with fewer than 100 variants, with HLA-A being the protein with the highest number of associated variants (3,624) (**Supplementary Figure 1f**).

In our main analysis, we used a p-value of 1e-03 as the threshold for including variants in the deep learning model. To better understand the effect of this threshold, we subsequently conducted a sensitivity analysis on a subset of 185 proteins. This subset included 138 proteins with identified non-linear effects and 47 randomly selected proteins across the full spectrum of model performance (i.e., R^2 0-0.1, 0.1-0.2, etc.). We re-ran our models using 5 different p-value thresholds: 1e-02, 1e-03 (original), 1e-04, 1e-05 and 1e-06. We found that the most stringent thresholds (1e-05 and 1e-06) resulted in some proteins having extremely few or even no variants to be modelled (not shown). The analysis of all 185 proteins was therefore conducted for the first 3 thresholds, while a subset of 117 proteins could be analyzed across all thresholds. We found that model performance generally improved with more stringent p-value cutoffs (**Supplementary Figure 2a,b,c,d**). For the 117 proteins, the mean R^2 performance increased from 0.267 (1e-02), to 0.279 (1e-03), 0.290 (1e-04) before plateauing at 0.294 for both 1e-05 and 1e-06. The decreased performance for the less stringent thresholds we found was likely due to overfitting from a larger number of input features. This analysis indicates that while our initial choice of 1e-03 yielded good results, an even better performance could have been achieved with a 1e-04 threshold. This demonstrates that there is an opportunity for enhancing the DL model performance further by using better tuned thresholds, or via a dynamic implementation that automatically tunes this parameter.

Supplementary Note 2: High modeling performance was associated with concordant Olink and SomaScan measurements

Multiple studies have compared antibody (Olink) and aptamer-based (SomaScan) proteomic profiling methods and found discrepancies in some protein measurements[4,5]. We, therefore, compared the correlation coefficients of Olink and SomaScan for 1,861 proteins from Eldjarn et al.[4], with the performance of our linear and DL models. We found that proteins with low performance in our models ($R^2<0.1$) had a low correlation between Olink and SomaScan (Wilcoxon rank-sum test, linear: W=326121, p=5.77e-14; DL: W=325089, p=1.511e-14) (**Supplementary Figure 3**). For example, of the proteins with $R^2<0.1$ using our DL model, 406 (21.8%) showed a Spearman correlation between Olink and SomaScan of <0.1, whereas only 182 (9.8%) of proteins with DL $R^2\geq0.1$ showed a Spearman correlation of <0.1 (**Supplementary Figure 3c**). This indicated that the low performance of some proteins in the DL and the linear models could be due to noisy protein measurements. Similarly, we investigated whether the type of Olink panel (Oncology, Neurology, Inflammation, Cardiometabolic) influenced the comparison between the DL and linear model. Here, we did not observe differences in performance gap (DL-linear) between the proteins from different Olink panels (**Supplementary Figure 3d**). However, we found that proteins from the first panels performed better using both DL and linear models than proteins from the second panels (t-test, two-sided, t = 17.109, df = 5739.9, p-value < 2.2e-16) (**Supplementary Figure 3e**). This was in line with results from Sun et al., who found a decreasing number of new associations with an increasing number of proteins measured[2].

Supplementary Figure 1

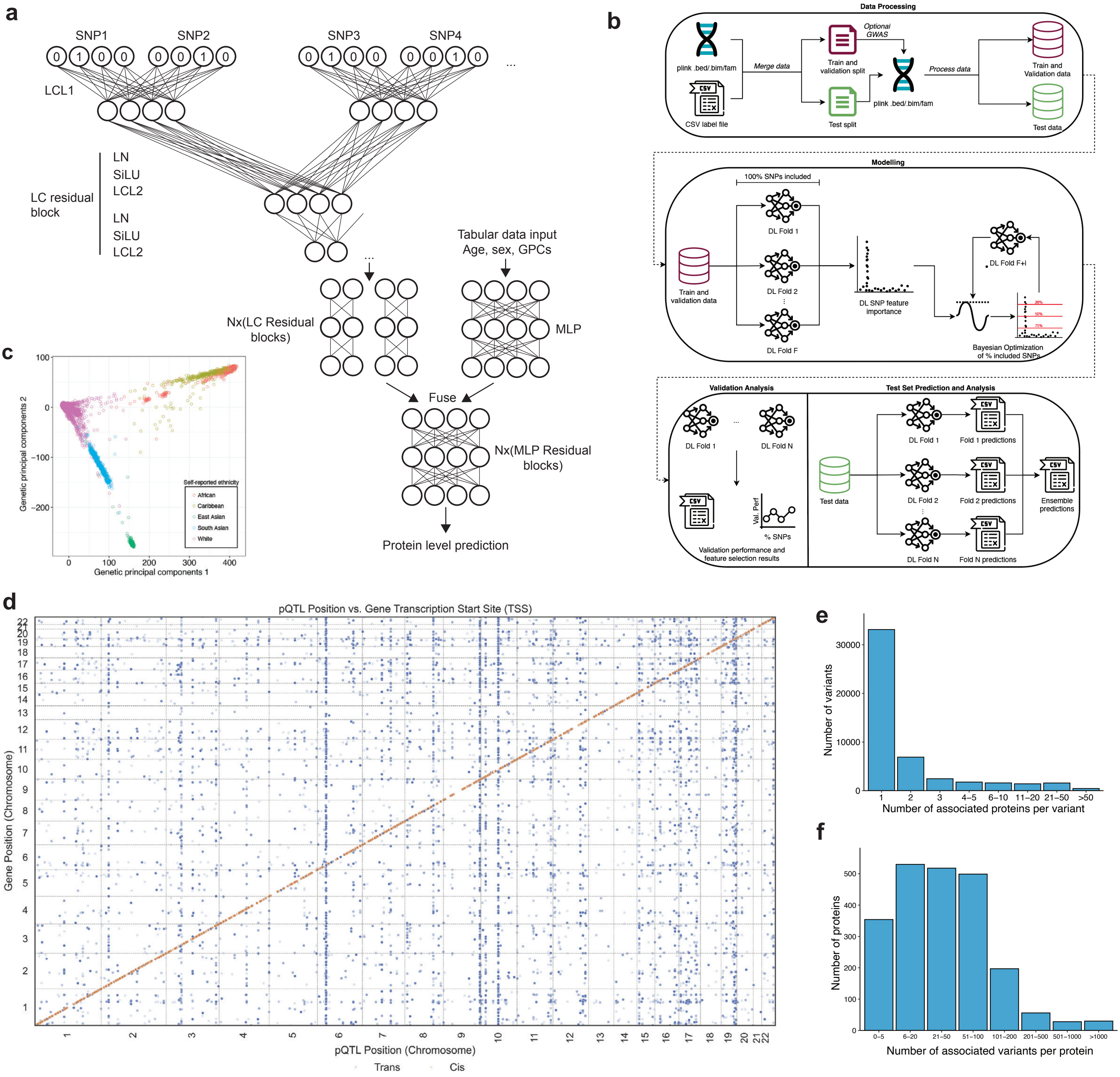

Supplementary Figure 1. a) Architecture of the Genome Local Net (GLN) as previously described in Sigurdsson et al., 2023[6]. **b)** Implementation of the GLN in the DL-framework EIR-auto-GP, which automates raw data processing, modeling, as well as validation and test set predictions. **c)** Genetic Principal Components for 52,700 participants in the UKB-PPP stratified by grouped self-reported ethnicities. **d)** pQTL and gene position of significant ($p<5e-11$) pQTL identified by GWAS for 2,922 proteins. Red indicates cis-pQTL and blue indicates trans-pQTL. **e)** Number of significantly associated proteins per variant identified by GWAS. **f)** Number of significant associations per protein identified by GWAS.

Supplementary Figure 2

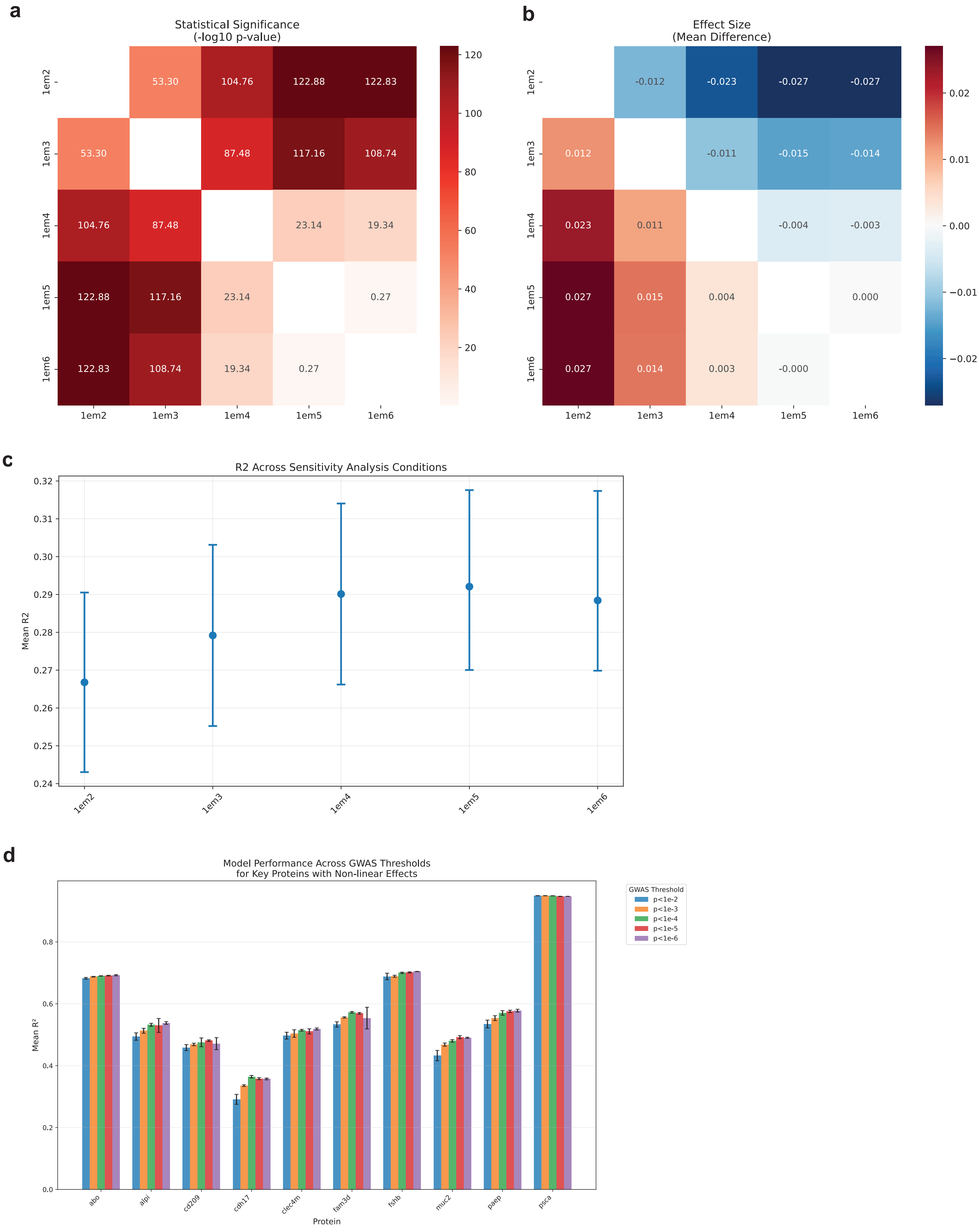

Supplementary Figure 2. a) Statistical significance heatmap showing pairwise comparisons between p-value thresholds using paired t-tests from sensitivity analysis testing five different GWAS p-value thresholds (1e-02, 1e-03, 1e-04, 1e-05, and 1e-06) on 117 proteins that could be successfully modelled across all thresholds. **b)** Effect size analysis for pairwise comparisons between p-value thresholds. **c)** Mean R^2 performance across the five p-value thresholds. Error bars represent the 95% confidence interval (mean ± 1.96 × SEM). Performance improved with more stringent thresholds, increasing from 0.267 (1e-02) to 0.279 (1e-03), 0.290 (1e-04), and plateauing at 0.294 for both 1e-05 and 1e-06. **d)** Individual protein performance across thresholds for selected proteins, showing the general trend of increased performance for proteins such as ALPI, FAM3D and MUC2. Error bars represent the standard deviation of R^2 values across the 5 folds.

Supplementary Figure 3

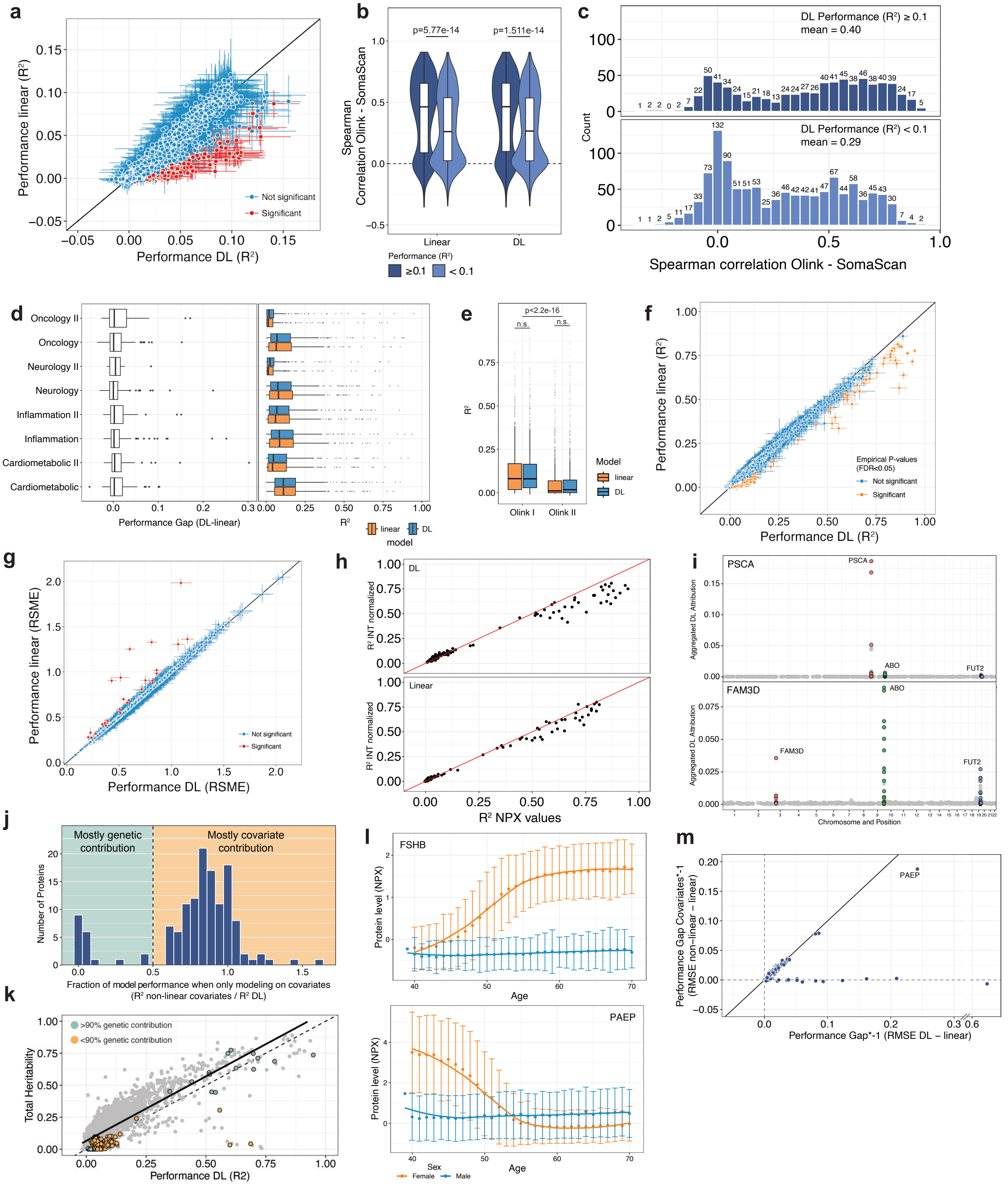

Supplementary Figure 3. **a)** DL and linear model performance for proteins with mean bootstrapped R^2 < 0.1 in DL or linear model. The error bars indicate the 95% confidence interval from 1,000 bootstraps and proteins with non-overlapping confidence intervals between DL and linear models were set as significant and labelled in red. **b)** Spearman correlation between SomaScan and Olink assay of 1,861 proteins from Eldjarn et al.[4] grouped by proteins with high ($R^2 \geq 0.1$) or low ($R^2 < 0.1$) performance in our DL or linear models. Statistical significance was calculated using the Wilcoxon rank-sum test. **c)** Histogram showing the distribution of proteins across Spearman correlation coefficient between SomaScan and Olink. The top panel shows proteins with high ($R^2 \geq 0.1$) DL performance, and the bottom panel shows proteins with low DL performance ($R^2 < 0.1$). **d)** Performance gap between DL and linear models (left) and the model performance (mean bootstrapped R^2) of DL and linear model (right) of proteins in 8 different panels of the Olink Explore assay. **e)** Model performance (mean bootstrapped R^2) of proteins in Olink panel **I** (Oncology **I**, Neurology **I**, Inflammation **I**, and Cardiometabolic **I**) or Panel **II** (Oncology **II**, Neurology **II**, Inflammation **II**, and Cardiometabolic **II**) in the DL or linear models. Statistical significance was calculated using a two-sided t-test. **f)** DL and linear model performance (R^2) of all 2,922 proteins. Proteins with significantly higher DL performance than linear model performance based on empirical P-values are labelled in orange. P-values were calculated using the bootstrapped distributions of R^2 for each protein as the proportion of bootstrap samples where the R^2 of DL was less than or equal to the R^2 of the linear model. Adjusted for multiple testing using the FDR method. **g)** DL and linear model performance (Root-mean-squared error (RMSE)) for all 2,922 proteins. The error bars indicate the 95% confidence interval from 1000 bootstraps and proteins with non-overlapping confidence intervals between DL and linear models are called significant and labelled in red. **h)** DL (top) and linear (bottom) model performance of 171 significant proteins modelled on raw protein expression values (NPX) or INT normalized protein values (**Supplementary Data 3**). **i)** Aggregated DL attribution of 642 and 757 SNVs across the genome that are used as input to model PSCA (top) and FAM3D (bottom) protein levels. Variants located within the three most activated loci are coloured and labelled. **j)** Number of proteins according to their fraction of performance (R^2) of non-linear model trained only on covariates compared to model performance of the DL model trained on covariates and genetics. If fraction is below 0.5, genetics is contributing most to the DL performance while fraction of more than 0.5 indicates mostly covariate contribution. **k)** Correlation of heritability estimates and DL model performance (R^2) with proteins (**Figure 2b**) labelled that showed more or less than 90% genetic contribution. 508 proteins without heritability estimates in Sun et al., were assigned total heritability of 0. **l)** Mean protein expression level (NPX) and standard deviation across different age groups (n=52,700) in the UKB stratified by sex (male, female) for FSHB (top) and PAEP (bottom). **m)** Performance gap (RMSE-RMSE) between DL and linear models on genotype and covariates against the performance gap between non-linear (XGBoost) and linear models on covariates only. Since lower RMSE indicates better performance, RMSE-based performance gaps were negated (i.e., multiplied by –1) to maintain interpretability and visual consistency with R^2-based comparisons.

Supplementary Figure 4

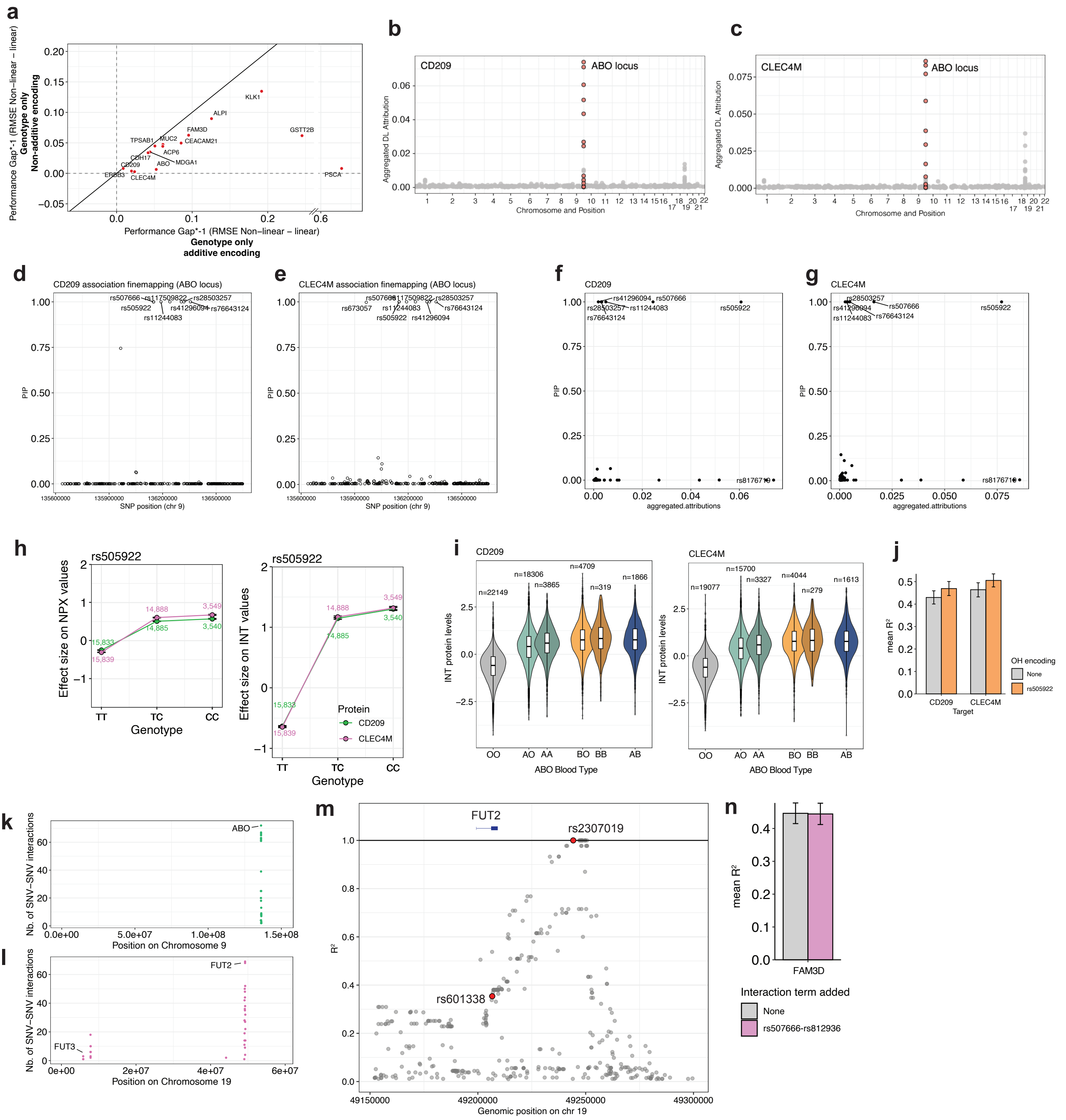

Supplementary Figure 4. a) Performance gap (RMSE-RMSE) between non-linear (XGBoost) and linear models trained and tested on additive or non-additive encoded genotype data for 15 candidate proteins with potential non-linear genetic effects. Since lower RMSE indicates better performance, RMSE-based performance gaps were negated (i.e., multiplied by –1) to maintain interpretability and visual consistency with R^2-based comparisons. **b)** Aggregated DL attribution of variants across the genome in the CD209 model. Variants located within the ABO locus (+/-500kb) are highlighted in red. **c)** Aggregated DL attribution of variants across the genome in the CLEC4M model. Variants located within the ABO locus (+/-500kb) are highlighted in red. **d)** Posterior inclusion probability (PIP) for 272 variants in the ABO locus (+/- 500kb) estimated by fine-mapping using pre-filtering GWAS summary statistics for CD209. Variants with high PIP (>0.9) are labelled. **e)** PIP for 272 variants in the ABO locus (+/- 500kb) estimated by fine-mapping using pre-filtering GWAS summary statistics for CLEC4M. Variants with high PIP (>0.9) are labelled. **f)** PIP correlation with aggregated DL attribution in the CD209 model. **g)** PIP correlation with aggregated DL attribution in the CLEC4M model. **h)** Effect size of different genotypes of *ABO* variant rs505922 on INT protein levels of CD209 and CLEC4M. Error bars indicate 95% confidence interval. Number of individuals with each genotype are indicated. **i)** INT protein levels of CD209 (n=51,214) in individuals in the UK Biobank, stratified by their imputed ABO blood group (field p23165)[7–10]. **j)** Linear model performance to predict CD209 and CLEC4M plasma levels trained on genotypes and covariates. One-hot encoded genotypes for rs505922 were added as single terms to assess performance improvement. Error bars indicate 95% confidence intervals of 1000 bootstraps. **k)** Number of interactions per unique SNV for variants on chromosome 9. Location of ABO locus is indicated. **l)** Number of interactions per unique SNV for variants on chromosome 19. Locations of FUT2 and FUT3 loci are indicated. **m)** Proxy variants and their R^2 are shown for variant rs2307019 (labelled) based on LDProxy[2,11] calculated for the British population (GBR). FUT2 rs601338 (Trp154Ter) that determines the FUT2 secretor status is labelled and the position of the FUT2 gene (NM_000511) is indicated above the plot. **n)** Linear model performance to predict FAM3D plasma levels trained on one-hot encoded genotypes and covariates. Interaction between rs507666 and rs812936 was added as a single term to assess performance improvement. Error bars indicate 95% confidence intervals of 1,000 bootstraps.

Supplementary Figure 5

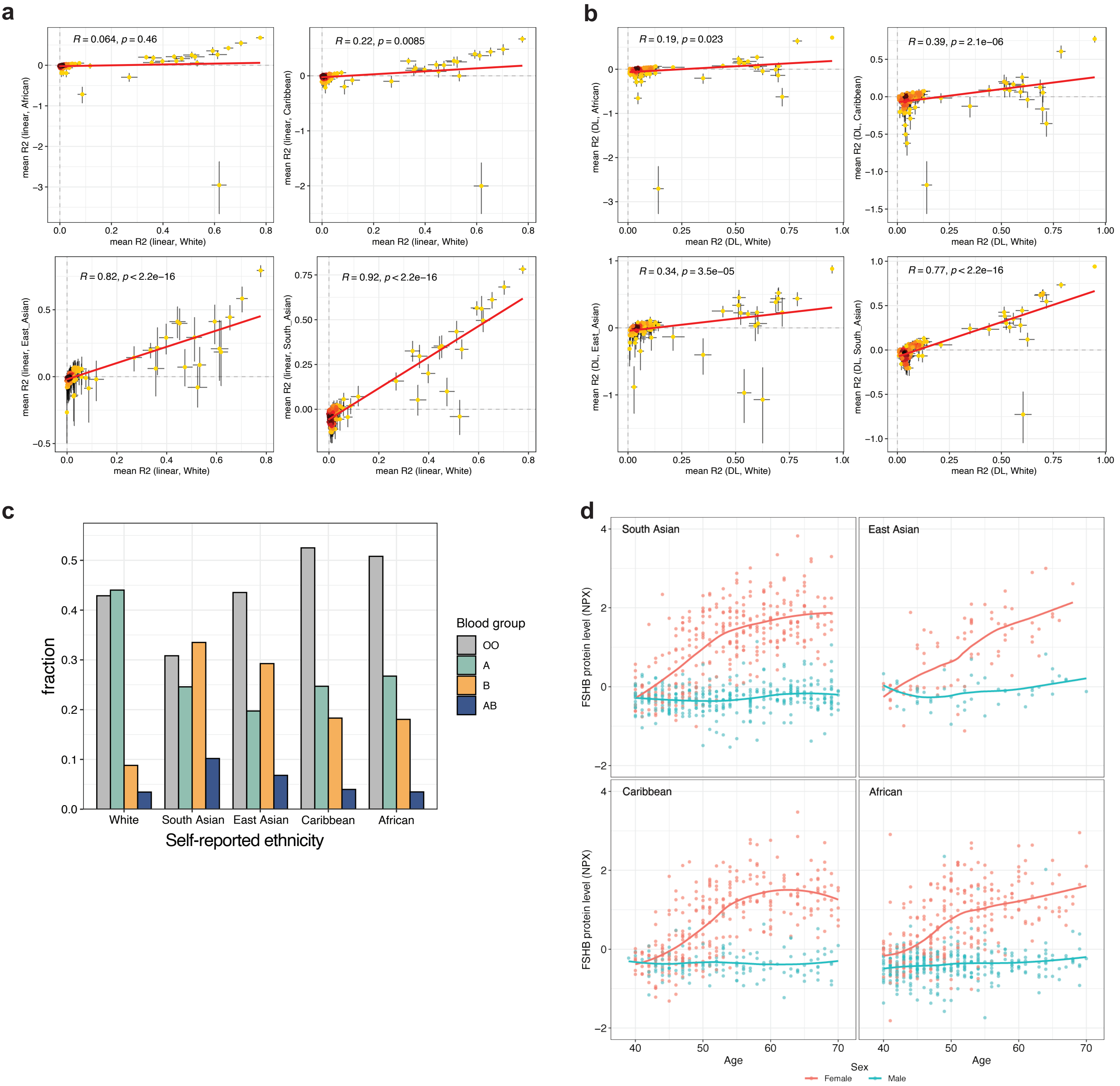

Supplementary Figure 5. a) Correlation of mean bootstrapped linear model performance (R^2) between test sets of White and African, Caribbean, East Asian or South Asian self-reported ethnicity. Results of Pearson correlation test are shown. Error bars indicate 95% confidence intervals of 1,000 bootstraps. **b)** Correlation of mean bootstrapped DL model performance (R^2) between test sets of White and African, Caribbean, East Asian or South Asian self-reported ethnicity. Results of Pearson correlation test is shown. Error bars indicate 95% confidence intervals of 1000 bootstraps. **c)** Distribution of imputed ABO blood groups within the self-reported ethnicity groups in the UKB-PPP. **d)** Age-dependent protein levels for FSHB between female and male individuals of South Asian, East Asian, Caribbean or African self-reported ethnicity test sets.

Supplementary Figure 6

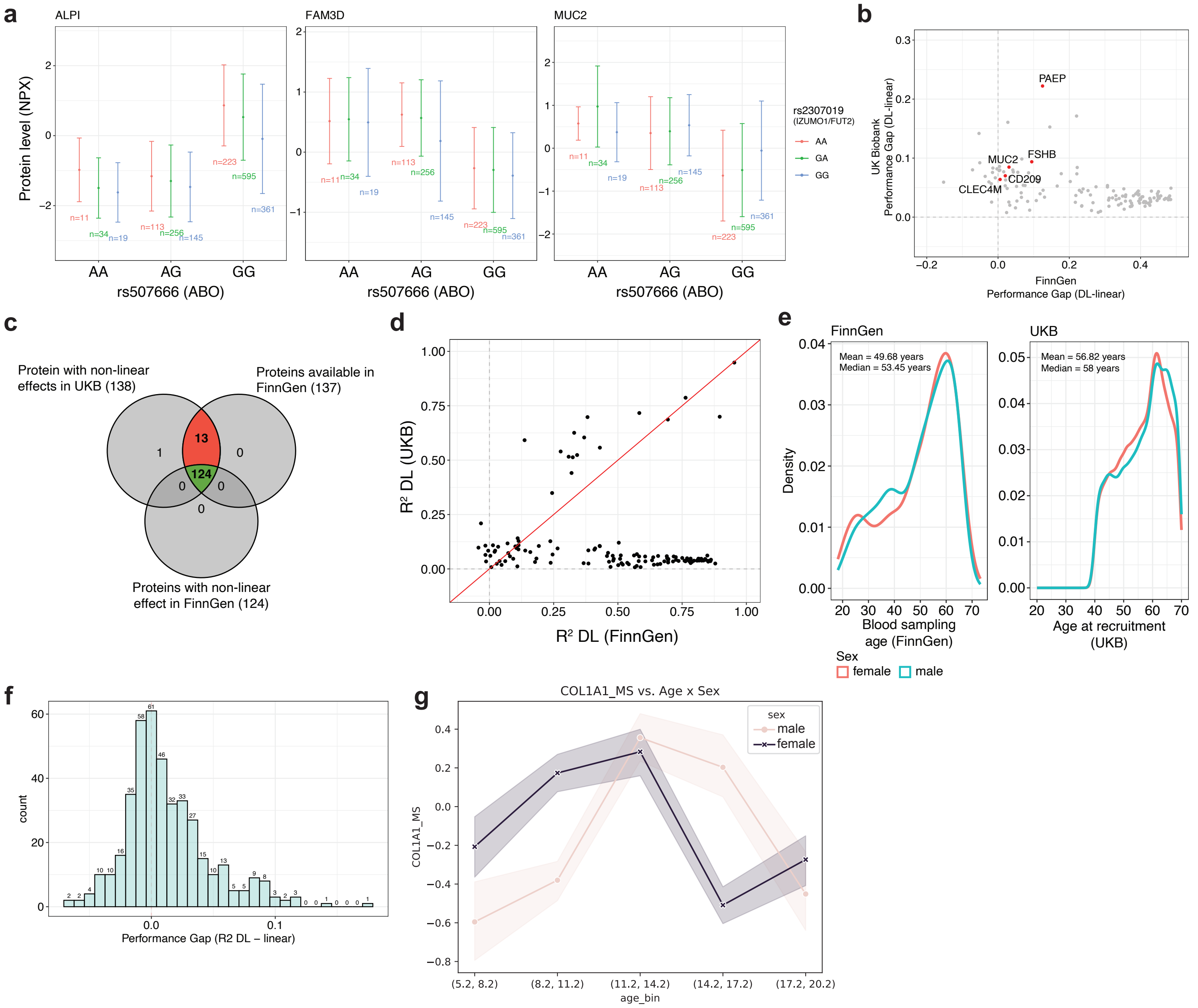

Supplementary Figure 6. a) Plasma levels (NPX) of ALPI, FAM3D and MUC2 in individuals from the FinnGen project in all combinations of genotypes of the *ABO* variant rs507666 and the *IZUMO1* variant rs2307019. Error bars indicate standard deviation. Numbers of individuals in the different genotype combinations are indicated. **b)** Correlation of Performance gap (R^2-R^2) between UKB and FinnGen for 137 proteins with non-linear effects identified in UKB showed replication of the non-linear covariate effects for FSHB and PAEP and potential non-linear genetic effects for MUC2, FAM3D and CD209. **c)** Venn diagram illustrating the replication success of non-linear effects in FinnGen. It shows the overlap between proteins with non-linear effects in the UKB, proteins modelled in FinnGen and the proteins with non-linear effects in FinnGen. **d)** Correlation of DL performance between UKB (mean bootstrapped R^2) and FinnGen (mean R^2) for 170 proteins. **e)** Distribution of the age at blood sampling stratified by sex in the FinnGen project (left) and distribution of the age at recruitment in 48,594 individuals in the UKB-PPP stratified by sex (right). Mean and median age of all participants in the respective cohort are indicated. **f)** Number of proteins and their distribution of performance (R^2) gap between DL (EIR) and linear (bigstatsr) model trained on 1,533 individuals and tested on 190 individuals from the Holbaek study. **g)** Plasma levels of COL1A1 in different age groups between 5.2 and 20.2 years stratified by sex.

Supplementary Table 1. Summary table of sample size in each ABO blood type group stratified by self-reported ethnicity. Combinations with <5 individuals were removed from the summary table. Related to Figure 4c.

	ABO blood type					
**Ethnicity**	**OO**	**AO**	**AA**	**BO**	**BB**	**AB**
**White**	21,048	17,808	3,803	4,111	210	1,688
**South Asian**	242	166	27	213	50	80
**East Asian**	64	27		36	7	10
**Caribbean**	267	102	16	88	9	19
**African**	350	158	17	114	15	25

Full membership of FinnGen Consortium

Aarno Palotie, Mark Daly, Bridget Riley-Gills, Howard Jacob, Coralie Viollet, Slavé Petrovski, Alix Berton, Santha Ramakrishnan, Ellen Tsai, Zhihao Ding, Emily Holzinger, Robert Plenge, Joseph Maranville, Mark McCarthy, Rion Pendergrass, Jonathan Davitte, Chia-Yen Chen, Melis Atalar Aksit, Anna Vlahiotis, Katherine Klinger, Clement Chatelain, Jorg Blankenstein, Karol Estrada, Robert Graham, Dawn Waterworth, Chris O´Donnell, Nicole Renaud, Tomi P. Mäkelä, Jaakko Kaprio, Minna Ruddock, Lila Kallio, Antti Hakanen, Terhi Kilpi, Markus Perola, Jukka Partanen, Taneli Raivio, Eero Punkka, Teija Kekonen, Raisa Serpi, Kati Kristiansson, Sanna Siltanen, Veli-Matti Kosma, Arto Mannermaa, Jari Laukkanen, Tiina Jokela, Mervi Ahlroth, Johanna Mäkelä, Outi Tuovila, Jeffrey Waring, Fedik Rahimov, Ioanna Tachmazidou, Marc Jung, Hanati Tuoken, Shameek Biswas, Benjamin Sun, Neha Raghavan, Jae-Hoon Sul, Xinli Hu, Ma´en Obeidat, Jonathan Chung, Jonas Zierer, Mari Niemi, Samuli Ripatti, Johanna Schleutker, Tiina Wahlfors, Mikko Arvas, Olli Carpén, Reetta Hinttala, Johannes Kettunen, Katriina Aalto-Setälä, Mika Kähönen, Hanna Kujala, Triin Laisk, Natalia Pujol, Veikko Salomaa, Jaana Suvisaari, Satu Koskela, Jouni Lauronen, Kristiina Aittomäki, Pirkko Pussinen, Tuomo Meretoja, Heikki Joensuu, Peeter Karihtala, Emma Juuri, Aino Salminen, Tuula Salo, David Rice, Pekka Nieminen, Ulla Palotie, Fredrik Åberg, Daniel Gordin, Patrik Finne, Joni A Turunen, Minna Raivio, Pentti Tienari, Martti Färkkilä, Jukka Koskela, Sampsa Pikkarainen, Kari Eklund, Paula Kauppi, Juha Sinisalo, Marja-Riitta Taskinen, Tiinamaija Tuomi, Timo Hiltunen, Johanna Mattson, Eveliina Salminen, Terhi Ollila, Katariina Hannula-Jouppi, Oskari Heikinheimo, Ilkka Kalliala, Lauri Aaltonen, Erkki Isometsä, Antti Aarnisalo, Ilkka Immonen, Salla Ranta, Filip Scheperjans, Felix Vaura, Nina Mars, Esa Pitkänen, Hannele Laivuori, Katja Kivinen, Elisabeth Widen, Taru Tukiainen, Hanna Ollila, Elmo Saarentaus, Anne Kerola, Eero Vuoksimaa, Joni Lindbohm, Zhiyu Yang, Matthew Sampson, Adrian Banerji, Michelle McNulty, Aoxing Liu, Joel Rämö, Austin Argentieri, Amanda Elliott, Elisa Rahikkala, Kirsi Sipilä, Valtteri Julkunen, Ville Leinonen, Sanna Toppila-Salmi, Mikko Hiltunen, Eino Solje, Hannu Kankaanranta, Antti Mäkitie, Iiris Hovatta, Niko Välimäki, Minttu Marttila, Anne Portaankorva, Eija Laakkonen, Heidi Silven, Eeva Sliz, Riikka Arffman, Susanna Savukoski, Riitta Kaarteenaho, Jaakko Tyrmi, Laura Kuusalo, Laura Pirilä, Tapio Hellman, Matti Vuori, Teemu Niiranen, Timo Blomster, Johanna Huhtakangas, Terttu Harju, Kaisa Tasanen, Laura Huilaja, Vuokko Anttonen, Marja Vääräsmäki, Outi Uimari, Laure Morin-Papunen, Maarit Niinimäki, Terhi Piltonen, Reetta Kälviäinen, Hilkka Soininen, Mikko Kiviniemi, Oili Kaipiainen-Seppänen, Margit Pelkonen, Päivi Auvinen, Maria Siponen, Liisa Suominen, Päivi Mäntylä, Kai Kaarniranta, Jukka Peltola, Airi Jussila, Katri Kaukinen, Pia Isomäki, Jussi Hernesniemi, Annika Auranen, Hannu Uusitalo, Teea Salmi, Venla Kurra, Laura Kotaniemi-Talonen, Argyro Bizaki-Vallaskangas, Juha Rinne, Roosa Kallionpää, Markku Voutilainen, Antti Palomäki, Riitta Lahesmaa, Kaj Metsärinne, Jenni Aittokallio, Klaus Elenius, Sirkku Peltonen, Leena Koulu, Ulvi Gursoy, Varpu Jokimaa, Tytti Willberg, Adam Ziemann, Nizar Smaoui, Anne Lehtonen, Apinya Lertratanakul, Relja Popovic, Mengzhen Liu, Anneke Den Hollander, Jan Freudenberg, Britney Milkovich, Andrew Blumenfeld, Tushar Kumar, Dirk Paul, Bram Prins, Eleanor Wheeler, Kousik Kundu, Santosh Atanur, Andrew Lowe, Thomas Spargo, Oliver Burren, Margarete Fabre, Fabio Baschiera, Hans van Leeuwen, Himanshu Manchanda, Karl Heilbron, Martin Rao, Nicole Schmidt, Samu Kurki, Johanna Mielke, Juho Immonen, Thomas Battram, Tobias Hogrebe, Susan Eaton, Ketian Yu, Stephanie Loomis, Coro Paisan-Ruiz, Elke Markert, Frank Li, Yao Hu, Christoph Ogris, Eric Simon, Julio Cesar Bolivar Lopez, Monika Frysz, Marla Hochfeld, Cara Carty, Michael Turchin, Neelakshi Jog, Corneliu Bodea, Janie Shelton, Chen Li, Kritika Singh, Peng Jiang, Elena Sanchez, Lilith Moss, Zijie Zhao, Anna Podgornaia, Natalie Bowers, Edmond Teng, Tim Lu, Hubert Chen, Jennifer Schutzman, Erich Strauss, Hao Chen, David Choy, Brian Yaspan, Cameron Adams, Michael Rothenberg, Sergio Dellepiane, Anubha Mahajan, Michael Holmes, Diana Chang, Tushar Bhangale, Fanli Xu, Laura Addis, John Eicher, Linda McCarthy, Jorge Esparza Gordillo, Joanna Betts, Rajashree Mishra, Audrey Chu, Diptee Kulkarni, Janet Kumar, Charli Harlow, Lea Sarow-Blat, Diana L.Cousminer, Jagtar Nijjar, Jessica Chao, Michal Magid, Shashank Jariwala, Chris Floyd, Dan Swerdlow, Erding Hu, Prerak Desai, Stephen Haddad, Damien Croteau-Chonka, Billy Fahy, Paola Bronson, Kirsi Auro, David Pulford, Sauli Vuoti, Dermot Reilly, Karen He, Ekaterina Khramtsova, Amy Hart, Meijian Guan, Alessandro Porello, P. Dunnmon, Sara Gale, Brice Keyes, John Kwon, Jonathan

Sherlock, Matt Loza, Chris Whelan, W Galpern, Yanfei Zhang, Mona Selej, Abolfazl Doostparast Torshizi, Qingqin S Li, Sahar Mozzafari, Christopher Deboever, Jason Miller, Fabiana Farias, Andrey Loboda, Jorge Del-aguila, Elisabeth Vollmann, Jozsef Karman, Julie Fiore, Rajesh Kamath, Andrei Popescu, Delphine Fagegaltier, Travis Barr, Aristide Merola, Oliver Freeman, Simonne Longerich, Enrico Ferrero, Nikos Patsopoulos, Nancy Finkel, Sabina Pfister, Shola Richards, Katherine Mccauley, Xiaobo Xia, Mike Mendelson, Majd Mouded, Debby Ngo, Kirsi Kalpala, Melissa Miller, Nan Bing, Jaakko Parkkinen, Heli Lehtonen, Stefan McDonough, Ying Wu, Erin Macdonald-Dunlop, Jessica Chung, Michael McLean, Joshua Chiou, Hye In Kim, Sivakumar Pitchumani, Sumedha Jassal, Madhurima Saxena, Catherine O'Riordan, Samuel Lessard, Suzanne Jacobs, Hamid Mattoo, David Habiel, Guanling Huan, Anu Jalanko, Risto Kajanne, Mervi Aavikko, Helen Cooper, Denise Öller, Tarja Laitinen, Sofia Kuitunen, Auli Toivola, Rodos Rodosthenous, Mitja Kurki, Juha Karjalainen, Pietro Della Briotta Parolo, Arto Lehisto, Juha Mehtonen, Reza Jabal, Mutaamba Maasha, Sanni Ruotsalainen, Samuel Jones, Raymond Walters, Paavo Häppölä, L. Elisa Lahtela, Johanna Paltta, Juulia Partanen, Mari Kaunisto, Elina Kilpeläinen, Tianduanyi Wang, Timo P. Sipilä, Oluwaseun Alexander Dada, Awaisa Ghazal, Rigbe Weldatsadik, Jaska Uimonen, Kati Donner, Anu Loukola, Päivi Laiho, Susanna Lemmelä, Teemu Paajanen, Arto Pietilä, Aki Havulinna, Mary Pat Reeve, Shanmukha Sampath Padmanabhuni, Harri Siirtola, Javier Gracia-Tabuenca, Marika Kaakinen, Shuang Luo, Vincent Llorens, Dawit Yohannes, Iina Laak, Pauli Wihuri, Tom Southerington, Meri Lähteenmäki

Supplementary References

1. Chang, C. C. *et al.* Second-generation PLINK: rising to the challenge of larger and richer datasets. *Gigascience* **4**, s13742-015-0047–8 (2015).
2. Sun, B. B. *et al.* Plasma proteomic associations with genetics and health in the UK Biobank. *Nature* **622**, 329–338 (2023).
3. Mbatchou, J. *et al.* Computationally efficient whole-genome regression for quantitative and binary traits. *Nat Genet* **53**, 1097–1103 (2021).
4. Eldjarn, G. H. *et al.* Large-scale plasma proteomics comparisons through genetics and disease associations. *Nature* **622**, 348–358 (2023).
5. Katz, D. H. *et al.* Proteomic profiling platforms head to head: Leveraging genetics and clinical traits to compare aptamer- and antibody-based methods. *Sci. Adv.* **8**, eabm5164 (2022).
6. Sigurdsson, A. I. *et al.* Deep integrative models for large-scale human genomics. *Nucleic Acids Research* **51**, e67–e67 (2023).
7. Melzer, D. *et al.* A Genome-Wide Association Study Identifies Protein Quantitative Trait Loci (pQTLs). *PLoS Genet* **4**, e1000072 (2008).
8. Paré, G. *et al.* Novel Association of ABO Histo-Blood Group Antigen with Soluble ICAM-1: Results of a Genome-Wide Association Study of 6,578 Women. *PLoS Genet* **4**, e1000118 (2008).
9. Wolpin, B. M. *et al.* Pancreatic Cancer Risk and ABO Blood Group Alleles: Results from the Pancreatic Cancer Cohort Consortium. *Cancer Research* **70**, 1015–1023 (2010).
10. Groot, H. E. *et al.* Genetically Determined ABO Blood Group and its Associations With Health and Disease. *ATVB* **40**, 830–838 (2020).
11. Machiela, M. J. & Chanock, S. J. LDlink: a web-based application for exploring population-specific haplotype structure and linking correlated alleles of possible functional variants. *Bioinformatics* **31**, 3555–3557 (2015).
