# Supplementary material for: Complex genetic effects linked to plasma protein abundance in the UK Biobank
Source: Nat Commun. 2025 Dec 14;17:533. doi: 10.1038/s41467-025-67235-0 (PMC12804929; doi:10.1038/s41467-025-67235-0)
Supplement: Supplementary file 2 — Description of Additional Supplementary Files [file 41467_2025_67235_MOESM2_ESM.pdf]

Description of Additional Supplementary Files

Supplementary Data 1. Combined summary statistics of the per-protein GWAS performed in this study. Related to Figure 1. (Available through https://doi.org/10.5281/zenodo.12654966)

Supplementary Data 2. Bootstrapped model performance for 2922 proteins on UK-white individuals in the test set (Olink batch 0-6). Related to Figure 2.

Supplementary Data 3. Model performance on INT normalized protein levels for 171 significant proteins. Related to Figure 2.

Supplementary Data 4. Bootstrapped model performance for 171 significant proteins on covariates only (age, sex, UKB batch, Consortium selected, first 20 genomic principal components) with non-linear (XGBoost) or linear model. Related to Figure 2.

Supplementary Data 5. Model performance for 171 significant proteins of non-linear (XGBoost) and linear models on additive and non-additive encoded genotype data. Related to Figure 2.

Supplementary Data 6. Significant SNV-SNV interactions for 15 proteins based on pair-wise OLS models. Related to Figure 3.

Supplementary Data 7. Bootstrapped model performance for 171 proteins tested on individuals of different self-reported ethnicities in the test dataset. Related to Figure 4.

Supplementary Data 8. Model performance for 137 proteins measured by Olink in the FinnGen cohort. Related to Figure 5.

Supplementary Data 9. Bootstrapped model performance for 411 proteins measured in the Holbaek cohort (tested on 190 individuals). Related to Figure 5.
