# Supplementary material for: Complex genetic effects linked to plasma protein abundance in the UK Biobank
Source: Nat Commun. 2025 Dec 14;17:533. doi: 10.1038/s41467-025-67235-0 (PMC12804929; doi:10.1038/s41467-025-67235-0)
Supplement: Supplementary file 4 — Reporting Summary [file 41467_2025_67235_MOESM4_ESM.pdf]

nature portfolio

Corresponding author(s): Simon Rasmussen

Last updated by author(s): Nov 3, 2025

Reporting Summary

Nature Portfolio wishes to improve the reproducibility of the work that we publish. This form provides structure for consistency and transparency in reporting. For further information on Nature Portfolio policies, see our Editorial Policies and the Editorial Policy Checklist.

Statistics

For all statistical analyses, confirm that the following items are present in the figure legend, table legend, main text, or Methods section.

n/a	Confirmed	
☐	☒	The exact sample size (*n*) for each experimental group/condition, given as a discrete number and unit of measurement
☒	☐	A statement on whether measurements were taken from distinct samples or whether the same sample was measured repeatedly
☐	☒	The statistical test(s) used AND whether they are one- or two-sided<br>*Only common tests should be described solely by name; describe more complex techniques in the Methods section.*
☐	☒	A description of all covariates tested
☐	☒	A description of any assumptions or corrections, such as tests of normality and adjustment for multiple comparisons
☐	☒	A full description of the statistical parameters including central tendency (e.g. means) or other basic estimates (e.g. regression coefficient) AND variation (e.g. standard deviation) or associated estimates of uncertainty (e.g. confidence intervals)
☐	☒	For null hypothesis testing, the test statistic (e.g. *F*, *t*, *r*) with confidence intervals, effect sizes, degrees of freedom and *P* value noted<br>*Give P values as exact values whenever suitable.*
☒	☐	For Bayesian analysis, information on the choice of priors and Markov chain Monte Carlo settings
☒	☐	For hierarchical and complex designs, identification of the appropriate level for tests and full reporting of outcomes
☐	☒	Estimates of effect sizes (e.g. Cohen's *d*, Pearson's *r*), indicating how they were calculated

Our web collection on statistics for biologists contains articles on many of the points above.

Software and code

Policy information about availability of computer code

Data collection	DNAnexus UKB-RAP
Data analysis	bigsnpr (1.12.2),<br>bigstatsr (1.5.12)<br>PLINK2 (v2.0.0-a.6.9)<br>PLINK v1.90b7<br>R (4.3.1)<br>tidyverse (2.0.0)<br>corrplot (0.95)<br>dplyr (1.1.4)<br>ggplot2 (3.5.2)<br>tibble (3.2.1)<br>ggpubr (0.6.0)<br>VennDiagram (1.7.3)<br>ggpointdensity (0.1.0)<br>tidyr (1.3.1)<br>susieR (v0.12.35)<br>EIR (https://github.com/arnor-sigurdsson/EIR)<br>EIR-auto-GP (commit fb41457, 0d5d762, c141b5a and 2934974, 0c627c0c; https://github.com/arnor-sigurdsson/EIR-auto-GP)

For manuscripts utilizing custom algorithms or software that are central to the research but not yet described in published literature, software must be made available to editors and reviewers. We strongly encourage code deposition in a community repository (e.g. GitHub). See the Nature Portfolio guidelines for submitting code & software for further information.

Data

Policy information about availability of data

All manuscripts must include a data availability statement. This statement should provide the following information, where applicable:

- Accession codes, unique identifiers, or web links for publicly available datasets
- A description of any restrictions on data availability
- For clinical datasets or third party data, please ensure that the statement adheres to our policy

UK Biobank genotype, proteomics and covariate data is available to approved researchers through the UK Biobank (https://www.ukbiobank.ac.uk/enable-your-research/apply-for-access). All analyses using UK Biobank data were performed at the Research Analysis Platform at DNAnexus (https://ukbiobank.dnanexus.com/). Combined summary statistics of the per-protein GWAS performed in this study (Supplementary Data) are available through Zenodo (https://doi.org/10.5281/zenodo.12654966). Individual-level genotypes and register data from FinnGen participants can be accessed by approved researchers via the Fingenious portal (https://site.fingenious.fi/en/) hosted by the Finnish Biobank Cooperative FinBB (https://finbb.fi/en/). Data release to FinBB is timed to the biannual public release of FinnGen summary results, which occurs 12 months after FinnGen consortium members can start working with the data. Data from The HOLBAEK Study is not publicly available due to the need to maintain privacy of study participants but is available on reasonable request. Searchable results are available online at proteomevariation.org.

Research involving human participants, their data, or biological material

Policy information about studies with human participants or human data. See also policy information about sex, gender (identity/presentation), and sexual orientation and race, ethnicity and racism.

Reporting on sex and gender	Sex was used as covariate throughout the study.<br>Individual-level information on a patients sex was taken from Data-field 31 in the UK Biobank.<br>Sex was acquired from central registry at recruitment, but in some cases updated by the participant. Hence this field may contain a mixture of the sex the NHS had recorded for the participant and self-reported sex (from UK Biobank).
Reporting on race, ethnicity, or other socially relevant groupings	Self-reported ethnicity was used in parts of the study to define training, validation and test sets and to test the trained models on data from different ethnic backgrounds.
Population characteristics	See Sun et al., 2023 (https://www.nature.com/articles/s41586-023-06592-6) for general characteristics of the UKB Pharma Proteomics Project. In our study, individuals from the COVID imaging study were excluded.
Recruitment	See UK Biobank
Ethics oversight	The UK Biobank has ethics approval from the North West - Haydock Research Ethics Committee.

Note that full information on the approval of the study protocol must also be provided in the manuscript.

Field-specific reporting

Please select the one below that is the best fit for your research. If you are not sure, read the appropriate sections before making your selection.

☒ Life sciences ☐ Behavioural & social sciences ☐ Ecological, evolutionary & environmental sciences

For a reference copy of the document with all sections, see nature.com/documents/nr-reporting-summary-flat.pdf

Life sciences study design

All studies must disclose on these points even when the disclosure is negative.

Sample size	Sample size was determined by data availability in the UK Biobank Pharma Proteomics Project.
Data exclusions	Individuals from the COVID-19 imaging study and related individuals were excluded from training, validation and test sets to create optimal conditions for model training. Additionally, all individuals from Olink batch 7 were removed due to increased variability (see Sun et al., 2023).
Replication	We replicated dominance effects of the ABO locus on CD209 and CLEC4M and epistatic effects between ABO and FUT2 locus on protein levels of ALPI, MUC2 and FAM3D in the FinnGen project. Additionally, we replicated our DL-based workflow in both FinnGen project and on MS-based proteomics data in the Holbaek Study to demonstrate the ability of the workflow to identify non-linear effects across cohorts and technology.
Randomization	Training, validation and test sets were not defined randomly. Instead, in the training dataset, we included exclusively individuals with self-reported 'UK-white' ethnicity from Olink batches 0-6. Batch 7 contains consortium selected individuals and individuals from the COVID-19 imaging study and do not follow UKB baseline characteristics. To this end, individuals from batch 7 and all individuals with non-UK-white ethnicity were excluded from the training dataset. Additionally, individuals from batch 7 were excluded in the UK-white test set (n=1,771) used throughout the study.

Blinding	This does not apply to our experimental setup.

Reporting for specific materials, systems and methods

We require information from authors about some types of materials, experimental systems and methods used in many studies. Here, indicate whether each material, system or method listed is relevant to your study. If you are not sure if a list item applies to your research, read the appropriate section before selecting a response.

Materials & experimental systems

n/a	Involved in the study
☒	☐ Antibodies
☒	☐ Eukaryotic cell lines
☒	☐ Palaeontology and archaeology
☒	☐ Animals and other organisms
☐	☒ Clinical data
☒	☐ Dual use research of concern
☒	☐ Plants

Methods

n/a	Involved in the study
☒	☐ ChIP-seq
☒	☐ Flow cytometry
☒	☐ MRI-based neuroimaging

Clinical data

Policy information about clinical studies

All manuscripts should comply with the ICMJE guidelines for publication of clinical research and a completed CONSORT checklist must be included with all submissions.

Clinical trial registration	UK Biobank (21/NW/0157); FinnGen study (HUS/990/2017), The HOLBAEK Study (NCT00928473)
Study protocol	UK Biobank (https://www.ukbiobank.ac.uk/media/gnkeyh2q/study-rationale.pdf), FinnGen study (10.1038/s41586-022-05473-8), The HOLBAEK Study (10.1371/journal.pone.0161921)
Data collection	-
Outcomes	-

Plants

Seed stocks	*Report on the source of all seed stocks or other plant material used. If applicable, state the seed stock centre and catalogue number. If plant specimens were collected from the field, describe the collection location, date and sampling procedures.*
Novel plant genotypes	*Describe the methods by which all novel plant genotypes were produced. This includes those generated by transgenic approaches, gene editing, chemical/radiation-based mutagenesis and hybridization. For transgenic lines, describe the transformation method, the number of independent lines analyzed and the generation upon which experiments were performed. For gene-edited lines, describe the editor used, the endogenous sequence targeted for editing, the targeting guide RNA sequence (if applicable) and how the editor was applied.*
Authentication	*Describe any authentication procedures for each seed stock used or novel genotype generated. Describe any experiments used to assess the effect of a mutation and, where applicable, how potential secondary effects (e.g. second site T-DNA insertions, mosiacism, off-target gene editing) were examined.*
